# Exploratory Untargeted Metabolomics of Dried Blood Spot Samples from Newborns with Maple Syrup Urine Disease

**DOI:** 10.3390/ijms25115720

**Published:** 2024-05-24

**Authors:** Abeer Z. Alotaibi, Reem H. AlMalki, Maha Al Mogren, Rajaa Sebaa, Mohammad Alanazi, Minnie Jacob, Ahamd Alodaib, Ahmad Alfares, Anas M. Abdel Rahman

**Affiliations:** 1 Genome Research Chair, Department of Biochemistry, College of Science, King Saud University, P.O. Box 22452, Riyadh 11652, Saudi Arabia; 441203289@student.ksu.edu.sa (A.Z.A.); msanazi@ksu.edu.sa (M.A.); 2 Metabolomics Section, Department of Clinical Genomics, Center for Genomics Medicine, King Faisal Specialist Hospital and Research Centre (KFSHRC), Riyadh 11211, Saudi Arabia; rgalmalki@kfshrc.edu.sa (R.H.A.); mmogren@kfshrc.edu.sa (M.A.M.); minnie@kfshrc.edu.sa (M.J.); aalodaib@kfshrc.edu.sa (A.A.); aalfares@kfshrc.edu.sa (A.A.); 3 Department of Medical Laboratories, College of Applied Medical Sciences, Shaqra University, Shaqra 11961, Saudi Arabia; r.sebaa@su.edu.sa; 4 Department of Biochemistry and Molecular Medicine, College of Medicine, Alfaisal University, Riyadh 11533, Saudi Arabia

**Keywords:** inborn errors of metabolism (IEMs), maple syrup urine disease (MSUD), genetic testing, newborn screening alloisoleucine, methionine sulfoxide, LysoPI, untargeted metabolomics, liquid chromatography high-resolution mass spectrometry

## Abstract

Currently, tandem mass spectrometry-based newborn screening (NBS), which examines targeted biomarkers, is the first approach used for the early detection of maple syrup urine disease (MSUD) in newborns, followed by confirmatory genetic mutation tests. However, these diagnostic approaches have limitations, demanding the development of additional tools for the diagnosis/screening of MUSD. Recently, untargeted metabolomics has been used to explore metabolic profiling and discover the potential biomarkers/pathways of inherited metabolic diseases. Thus, we aimed to discover a distinctive metabolic profile and biomarkers/pathways for MSUD newborns using untargeted metabolomics. Herein, untargeted metabolomics was used to analyze dried blood spot (DBS) samples from 22 MSUD and 22 healthy control newborns. Our data identified 210 altered endogenous metabolites in MSUD newborns and new potential MSUD biomarkers, particularly L-alloisoleucine, methionine, and lysoPI. In addition, the most impacted pathways in MSUD newborns were the ascorbate and aldarate pathways and pentose and glucuronate interconversions, suggesting that oxidative and detoxification events may occur in early life. Our approach leads to the identification of new potential biomarkers/pathways that could be used for the early diagnosis/screening of MSUD newborns but require further validation studies. Our untargeted metabolomics findings have undoubtedly added new insights to our understanding of the pathogenicity of MSUD, which helps us select the appropriate early treatments for better health outcomes.

## 1. Introduction

Clinically, inborn errors of metabolism (IEMs) are a group of rare heterogeneous disorders genetically transmitted through autosomal or X-linked recessive inheritance patterns. These genetic disorders are associated with accumulations of intermediary metabolites, with subsequent shortages of metabolic products, leading to pathological consequences associated with various phenotypes [1,2]. Based on IEMbase, almost 1872 IEMs have been identified, and more disorders are expected to be discovered (http://www.iembase.org/, accessed on 2 December 2022) [3]. In particular, maple syrup urine disease (MSUD), known as an aminoacidopathy, is one of the categorized IEMs caused by a deficiency of the mitochondrial enzyme called the branched-chain ketoacid dehydrogenase (BCKD) complex, resulting from mutations in the genes that encode complex BCKD subunits including E1α, E1β, E2, and E3 [4]. According to the Human Gene Mutation Database, 259 genetic mutations have been reported to cause MSUD (http://www.hgmd.cf.ac.uk/ac/index.php, accessed on 1 October 2023). Imtiaz et al. reported, in 2017, 20 novel MSUD-related genetic mutations in *BCKDHA*, *BCKDHB*, and *DBT* in Saudi patients [5]. Generally, MSUD patients are classified into classic MSUD, accounting for 80% of the cases developed in newborns after two days of birth, and milder variants of MSUD, i.e., intermediate and intermittent cases, detected at older ages [6]. Phenotypically, MSUD patients vary in their symptoms based on their disease type, as classical MSUD has the most severe symptoms, including hypotonia, severe vomiting, seizures with encephalopathy, coma, and brain edema, while the milder variants of MSUD have lesser degrees of these clinical symptoms [7,8]. Pathologically, MSUD patients are characterized by derangements at the phenotypic, genetic, and metabolic levels. Briefly, MSUD-related genetic mutations lead to significant metabolic alterations, specifically, elevations of branched-chain amino acids (BCAAs)—including leucine, isoleucine, alloisoleucine, and valine—and branched-chain α-ketoacids (BCKAs) in the body tissues and fluids of MSUD patients. These metabolic alterations may not be seen in all types of MSUD [9]. However, among these metabolic alterations, alloisoleucine, which is derived from isoleucine in vivo by transamination, is elevated in all MSUD patient types and thus is used as a pathogenic marker for MSUD [10].

The global incidence of MSUD is estimated to be 1:185,000, affecting all ethnic populations. In Saudi Arabia, the frequency of MSUD is not well known; however, based on newborn screening (NBS), it is estimated to be 1:21,490 [5]. As expected, this prevalence is underestimated due to the possible existence of undiagnosed MSUD patients and false-positive cases [11,12], which lead to high demands for treatments and interventions that negatively affect the health system and cause financial burdens, encouraging the improvement of the accuracy of MSUD’s identification in early life. The early and confirmed identification of MSUD during the neonatal period is achieved based on MSUD-related clinical phenotypes and manifestations: elevations of BCAA and BCKA detected through NBS and the detection of BCKD gene mutations via genetic testing. To further illustrate this, NBS analyses of MSUD rely on the tandem mass spectrometry (MS/MS) analysis of dried blood spots (DBSs) taken from newborns’ heels to measure targeted metabolic biomarkers and their concentrations, particularly “total Leu” (Xle), which encompasses leucine (Leu), isoleucine (Ile), valine, and alloisoleucine (Allo-Ile) [10,11]. If the NBS results are positive, then follow-up genetic tests are performed using high-throughput genomic sequencing combined with Sanger sequencing for their confirmation and validation [12]. Notably, NBS and genetic testing have advanced the identification of MSUD cases [13,14], but these approaches have certain limitations that must be resolved. Additionally, genetic testing can be expensive, is occasionally accompanied by delayed results, and detects variants of uncertain significance (VUSs) [15,16]. The last-mentioned variant (VUS) requires functional analyses to examine its impact and relation to the MSUD condition. In addition to the genetic testing-related limitations, NBS also faces some obstacles. NBS can detect newborns who do not truly have MSUD (false-positive cases) or miss cases for unknown reasons. To improve the accuracy and sensitivity of both approaches and solve their limitations, additional strategies are required to diagnose and screen for true MSUD cases in early life, and these could be applied in parallel with the currently used approaches. Currently, the clinical and scientific communities have recommended utilizing untargeted metabolomics as a tool for identifying IEMs, including MSUD, for the following reasons: Firstly, since metabolism and health are strongly linked, untargeted metabolomics, which measures a wide range of small metabolites representing the metabolism status, and detects any metabolic alterations can provide valuable insights into healthy and pathological conditions.

Furthermore, untargeted metabolomics relatively measures the amounts of metabolites, the final byproducts of genes involved in metabolic pathways. According to the Human Metabolome Database (HMDB), approximately thousands of metabolites have been found [17]. These metabolites could be used as potential biomarkers for IEMs diseases. Most importantly, the great opportunity of untargeted metabolomics is that it provides comprehensive coverage of metabolites in the body system, which is in opposition to the limitations of the current biochemical or metabolic tests, which measure a limited number of metabolites, leading to them missing other altered metabolites with a significant ability to be used as disease-specific markers. Promisingly, using untargeted metabolomics can solve these issues seen with genetic testing and NBS and bridge the knowledge gap in IEMs [18].

To date, a few studies have used untargeted metabolomics in MSUD patients. In 2018, Coene et al. performed an untargeted metabolomic profiling of two MSUD patients included in their research, finding that leucine, isoleucine, ketoleucine, 2-hydroxymethyl butyric acid, and 2-hydroxy caproic acid were dysregulated significantly compared to their healthy population [19]. Another untargeted metabolomics research study performed on LC-QTOF for 21 MSUD infants revealed elevations of 3-hydroxybutyric acid, 2-oxoisovaleric acid, 2-hydroxyisovaleric acid, 2-oxo-3-methylvaleric acid, total leucine (XLeu), valine (Val), and others [20]. Furthermore, Haijes et al. (2019) evaluated seven MSUD patients’ plasma, and they found that their isoleucine and alloisoleucine with keto-acids were elevated, while their 3-hydroxyisobutyrate and isovaleryl-carnitine were reduced [21]. These published findings illustrate the promising potential of using metabolomics as a diagnosis/screening tool for MSUD; however, these studies have some limitations, such as a low number of MSUD participants of various ages and the use of different analytical instruments to perform their metabolomics analyses. Thus, further metabolomics studies are required to validate their findings; to explore additional metabolic biomarkers of MSUD, especially in the neonatal period; and to standardize the workflow of metabolomics analyses by using a larger cohort of MSUD newborns, which could lead more accuracy in the early diagnosis of MSUD. Thus, we aimed to conduct an untargeted metabolomics analysis of DBS samples from MSUD newborns to discover the metabolic biomarkers and pathways that could improve the inaccurate diagnoses of MSUD caused by heterogeneous MSUD phenotypes, false NBS results, and VUS cases.

## 2. Results

### 2.1. Participant Demographics and DBS Sample Selection

In order to use appropriate samples in our study, DBS samples from newborns that were admitted to the NBS lab at King Faisal Specialized Hospital and Research Center (KFSHRC) were first checked and identified as either MSUD samples (according to the NBS-based-MSUD markers mentioned below) or normal samples showing normal NBS results. A total of 44 DBS samples were used in this study: 22 samples were collected from biochemically and genetically confirmed MSUD newborns, and the other 22 samples were age- and gender-matched healthy newborns (Table 1). The participants in the two groups were almost age- and gender-matched. The average age of the MSUD newborns was (7.63 ± 3.07 days) and for healthy newborns was (7.72 ± 3.04 days). Newborns older than 14 days or with other IEMs, based on their NBS data, were excluded from this study. MSUD newborns showed significantly increased Xleucine (581 ± 431.97 µM) and valine (431.97 ± 149.02 µM) compared to healthy newborns. For the NBS data, DBS samples from these newborns were used for untargeted metabolomics analyses.

### 2.2. Untargeted Metabolomics Profiling of MSUD Newborns

A total of 28,769 *m*/*z* compound ions were detected (Table S1) in both positive (*n* = 19,336) and negative (*n* = 9433) ionization modes. The data were deposited in Metabolomics Workbench (ST002750). In total, 28.5% of missing values were excluded after applying a filter with a frequency > 80% to ensure quality in the data analyses, resulting in 20,568 features being retained for statistical analysis. To confirm that all depicted data have a Gaussian distribution, the median was identified, we log-transformed normalized the data, and the data were Pareto scaled to eliminate systemic variances.

**Figure 1:**
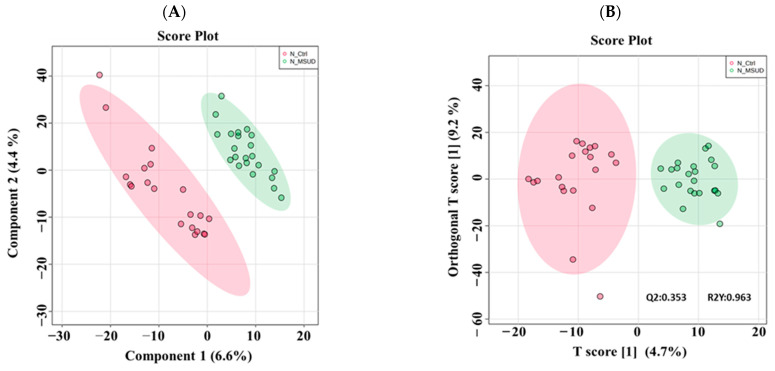
Sample clustering and group separation based on a group of 20,568 features. (**A**) PLS-DA shows a clear separation between the two groups: MSUD newborn and healthy control. (**B**) OPLS-DA shows a clear separation between the two groups: MSUD newborns and healthy controls. The robustness of the created models was evaluated by the fitness of the model (R^2^Y: 0.963) and predictive ability (Q^2^: 0.353) values.

A multivariate analysis using partial least squares discriminant analysis (PLS-DA), a non-supervised analysis, showed sample clustering and a clear separation between MSUD newborns (green) and healthy controls (red; Figure 1A). Additionally, orthogonal partial least squares discriminant analysis (OPLS-DA), a supervised analysis, was performed and showed a clear separation in the score plot, reflecting the difference between the two groups, with a computed R^2^Y = 0.963 and Q^2^ = 0.353, as shown in Figure 1B.

**Figure 2:**
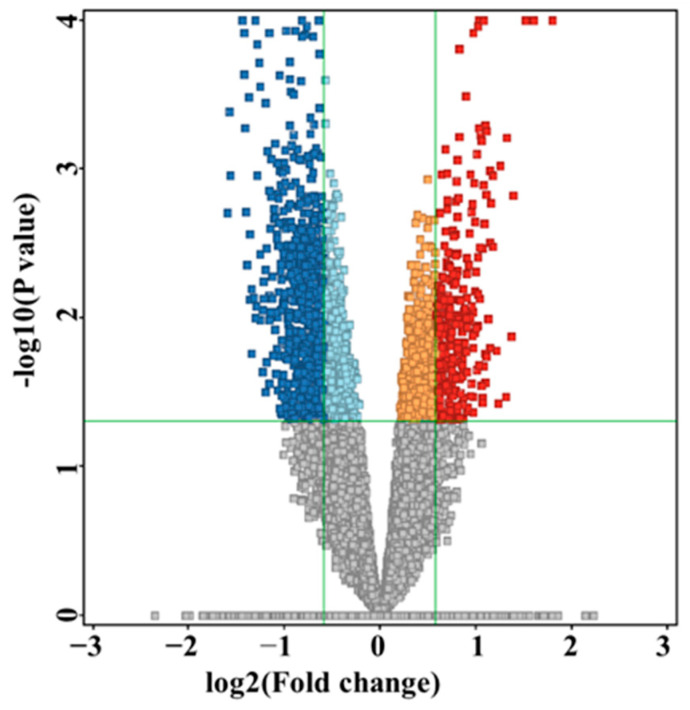
Volcano plot (moderated *t*-test, cut-off: *p* < 0.05, FC 1.5) between two groups: MSUD newborns and healthy controls. The heatmap revealed 1040 significantly dysregulated metabolites, where 303 (red) and 737 (blue) were up- and downregulated, respectively.

A univariate analysis was performed after normalizing the signal and ensuring normal distribution. A volcano plot (moderated *t*-test, cut-off *p*-value ≤ 0.05 and fold change 1.5) was used to identify the significantly altered features between the two groups, MSUD newborns and healthy controls, and revealed 1040 significantly dysregulated features, where 303 and 737 were up- and downregulated, respectively (Figure 2; Table S2). A total of 480 features were annotated using HMDB (Table S3), and 210 of these were identified as human endogenous metabolites after excluding the exogenous molecules (i.e., environmental exposure, drugs, foods, etc.); these are listed in Table S4.

**Figure 3:**
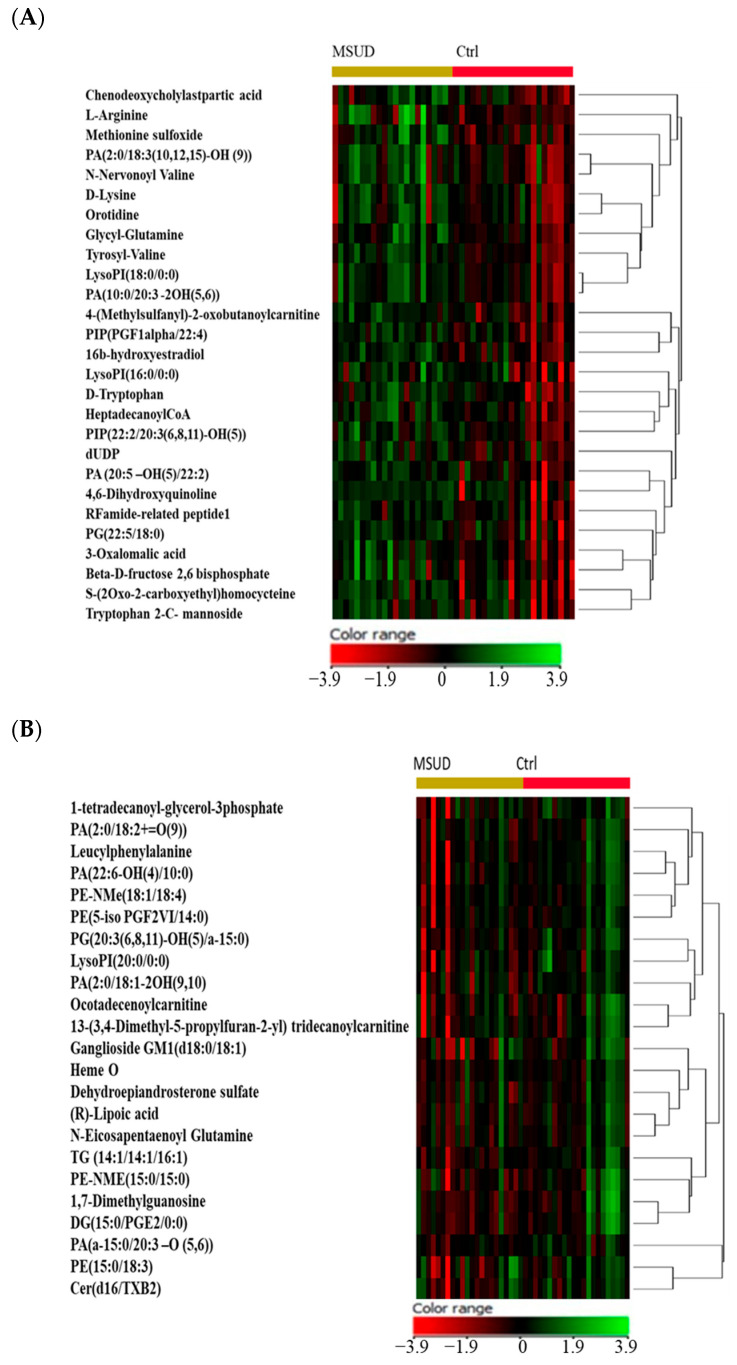
Hierarchical clustering (HAC) and heatmap analyses demonstrating (**A**) Upregulated metabolites. (**B**) Downregulated metabolites in MSUD newborns compared with healthy controls. The color scaled bar, green referred to upregulated metabolites, and red referred to downregulated metabolites.

A heatmap was constructed that identified 210 as endogenous metabolites, of which 51 and 159 significantly were up- and downregulated in MSUD newborns (Figure S1; Figure 3A and 3B), respectively.

### 2.3. Biomarkers Analysis of MSUD

**Figure 4:**
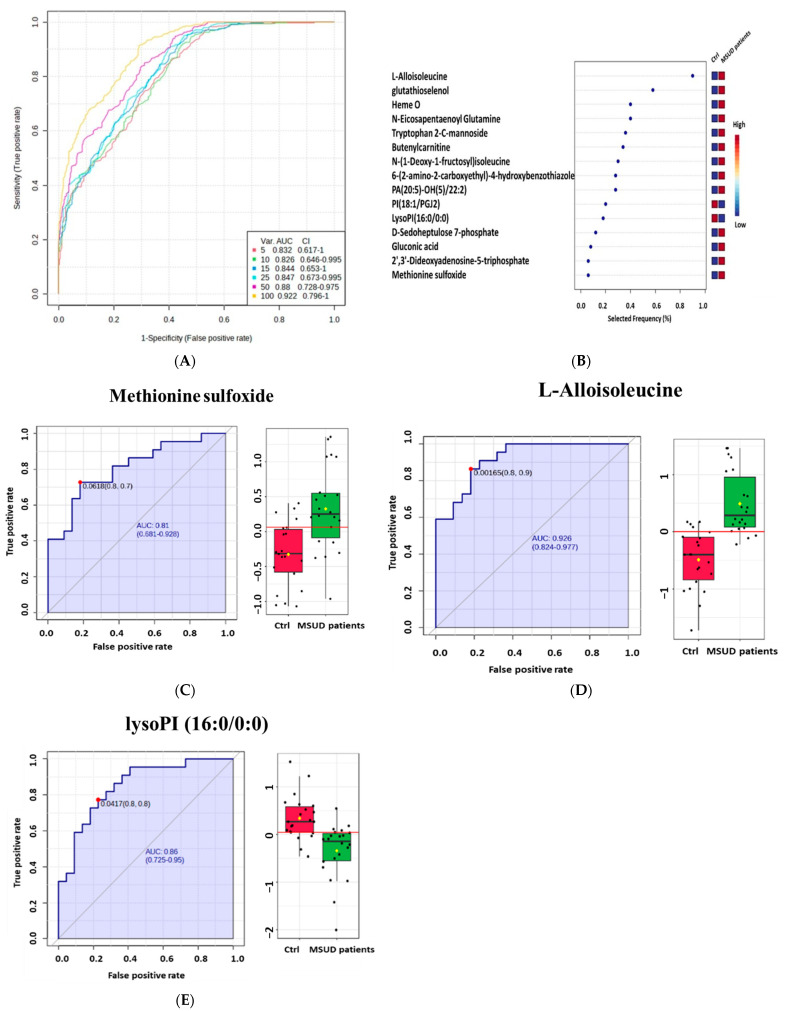
Metabolomics profiling and biomarker evaluation between MSUD newborn and healthy control groups. (**A**): A receiver operating characteristics (ROC) curve was created by the OPLS-DA model, with area under the curve (AUC) values calculated from the combination of 5, 10, 15, 25, 50, and 100 metabolites (**B**): The frequency plot shows the top 15 identified metabolites. (**C**,**D**): Examples of metabolites methionine sulfoxide and L-alloisoleucine were upregulated in MSUD newborn patients with (AUC:0.81) and (AUC: 0.926), respectively. (**E**): lysoPI downregulated in MSUD newborns compared to healthy control (AUC: 0.86).

MSUD biomarkers were evaluated using a receiver operating characteristics (ROC) curve analysis. As a classification and feature, the ranking approach PLS-DA was used to create a multivariate exploratory ROC analysis. Six features of the ROC curve via PLS-DA and cross-validation (CV) had area under the curve (AUC) values ranging from 0.832 to 0.922 with confidence intervals of 0.617–1 and 0.796–1 (Figure 4A). The frequency plot illustrates the 15 highest-scoring identified metabolites in the OPLS-DA model according to their level in MSUD and healthy newborns. As shown, L-alloisoleucine, methionine sulfoxide, glutathioselenol, heme O, N-eicosapentaenoyl glutamine, tryptophan 2-C-mannosidase, butenyl carnitine, and N-(1-Deoxy-1-fructosyl) isoleucine were upregulated endogenous metabolites. At the same time, PI (18:1/PGJ2) and lysoPI (16:0/0:0) were downregulated metabolites in MSUD newborns compared to the healthy control group (Figure 4B). For example, the AUC value of the ROC curves for methionine sulfoxide (Figure 4C) and L-alloisoleucine (Figure 4D) were upregulated (AUC: 0.81) and (AUC: 0.926), respectively. LysoPI was downregulated in MSUD newborns compared to their corresponding healthy controls (AUC: 0.86).

### 2.4. Metabolomic Pathway Analysis

**Figure 5:**
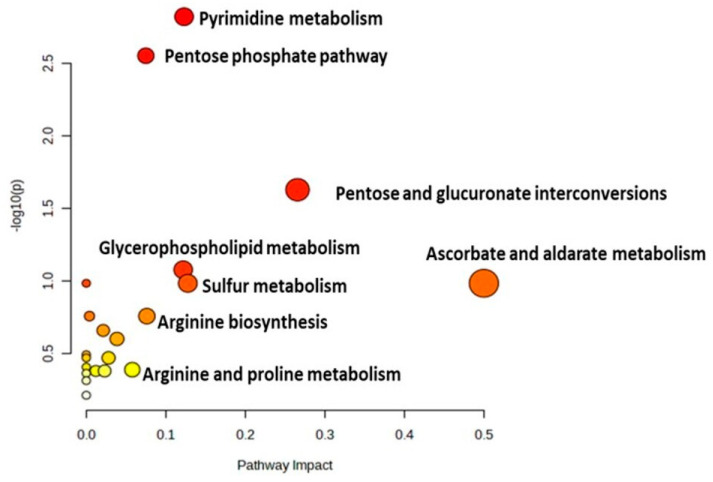
Pathway analysis of the significant metabolites dysregulated in MSUD newborns. In total, 210 metabolites were finally identified as human endogenous metabolites; 51 were upregulated, and 159 were downregulated. The color variation (yellow to red) shows the different significance levels of metabolites in the data.

All these altered metabolites were subjected to a pathway analysis to identify the most affected pathways between the two study groups, which were ascorbate and aldarate metabolism and pentose and glucuronate interconversions. Other pathways were affected, such as sulfur metabolism, pyrimidine metabolism, glycerophospholipid metabolism, and pentose phosphate pathways, as illustrated in Figure 5 and detailed in Table S5.

## 3. Discussion

### 3.1. Untargeted Metabolomics as an Additional Diagnostic/Screening Tool for MSUD

In the clinical field, the early identification of MSUD is much preferred to avoid health complications and manage disease progression by providing MSUD newborns with the proper treatment in their neonatal period. Thus, MSUD diagnosis needs to be more accurate in early life. Currently, MSUD is diagnosed based on certain clinical criteria, including phenotypes/symptoms, NBS results, and genetic testing [2]. However, the last-mentioned clinical criteria have limitations, causing inaccuracy in the diagnosis of MSUD. For example, MSUD patients exhibit variable and heterogeneous phenotypes/symptoms, which cause difficulties in their early diagnosis.

Furthermore, NBS and genetic testing approaches have limited diagnostic/screening abilities in some MSUD cases, and NBS has previously led to false-positive or -negative cases of MSUD. For example, negative cases of MSUD have been seen, with samples collected from newborns at 24 h of life [22]. On the other hand, false-positive cases of MSUD have been detected in newborns diagnosed with hydroxyprolinemia, in which hydroxyproline is not distinguished from leucine and isoleucine by low-resolution mass spectrometry [23]. Additionally, during pregnancy, the maternal ingestion of sotolone-containing foods such as fenugreek can lead to false-positive cases of MSUD in newborns [24]. In addition to the limitations of NBS, genetic testing may detect VUS; the latter is detected in these analyses but does not provide detailed information about the mutation, whether disease-related or not, and further deep functional studies are required to identify the impact and relationship of VUS to the disease [25]. Some reports illustrate that some newly identified MSUD patients are found to have VUS in their MSUD-related genetic mutations and require functional analyses to confirm the pathogenicity of these variants [26,27].

Therefore, there have been increasing demands and attempts in the scientific community to solve these limitations by developing a new diagnostic tool for the better diagnosis of MSUD. Notably, untargeted metabolomics has received a lot of attention as a newly emerged diagnostic tool for IMDs because it can explore their wide metabolic profile, reflecting the metabolic status of diseases, which gives us a better understanding of IMDs. Additionally, untargeted metabolomics can uncover new metabolic biomarkers and pathways that could be added to the clinical diagnostic criteria for IMDs, improving their overall diagnosis process and increasing accuracy. A few studies have been carried out on the metabolomics of different MSUD samples, including DBS and urine, which were analyzed using MS or NMR methods [28,29,30]. Still, no study has performed untargeted metabolomics analyses of samples from MSUD newborns to identify its metabolic alterations and biomarkers/pathways in early life. Therefore, we performed untargeted metabolomics analyses of DBS samples from MSUD newborns, and our metabolomics results showed that MSUD newborns have an altered metabolic profile compared to healthy newborns (controls). Additionally, our findings revealed potential metabolic biomarkers and pathways that could be used for MSUD diagnosis/screening in early life, which are discussed further below.

### 3.2. Untargeted Metabolomics Revealed Altered Global Metabolic Profiling of MSUD Newborns

To truly study the metabolic alterations caused by this disease, we aimed to comprehensively explore the metabolic profiling of MSUD newborns who were not exposed to external factors, such as treatments or diet interventions, because the latter could modulate the true metabolic status of MSUD samples. Therefore, DBS samples were taken from MSUD newborns of an average age of around 7 days and analyzed using untargeted metabolomics. Our untargeted metabolomics analyses of MSUD DBSs revealed that MSUD had an altered metabolic profile compared to healthy newborns, with a broader range of altered metabolites. Examples of these altered metabolites include amino acids, lipid species, purine derivatives, glucuronide conjugates, and oxidative-related compounds.

### 3.3. Alterations in the Amino Acids in MSUD Newborns

Our data clearly showed that DBS samples from MSUD contained various altered amino acids and their modified forms, such as tryptophan, methionine, leucine, and alloisoleucine. For instance, tryptophan 2-C-mannoside was upregulated in MSUD newborns compared to healthy newborns. Tryptophan 2-C-mannoside, known as mannosyl tryptophan (CMW), is a glycosylated form of tryptophan in which the c-mannosylation site is found in the tryptophan motif located in proteins when it is targeted, it causes important protein folding, sorting, and/or secretion [31,32]. Interestingly, CMW levels were increased in the blood of patients with renal dysfunction, and they were also found to be elevated in patients with T2D [33], suggesting that CMW could act as a pathological biomarker in diseases. Given our CMW findings and the previously published studies on MCW, it is possible that CMW is elevated in various pathological conditions, including MSUD. However, further investigations are needed to deeply explore the role of MCW in the complication or progression of MSUD.

Additionally, our data revealed that methionine sulfoxide was upregulated in MSUD newborns compared to healthy controls. Methionine sulfoxide is derived from methionine and is formed post-translationally through the oxidation of methionine sulfur [34]. Methionine sulfoxide is an important metabolite that affects redox homeostasis in diseases through sulfur metabolism [35,36]. It is possible that MSUD patients could be subject to certain oxidative stress events, as is partially indicated by their elevated levels of methionine sulfoxide, which in turn could lead to the neurodegeneration and functional impairment of cells known to occur in MSUD [37]. It is suggested that methionine sulfoxide could be targeted and returned to methionine to potentially reduce some of the oxidative and degradative damages found in MSUD through reversing this post-translation modification using targeted-site modification strategies, especially as methionine is known to have antioxidant properties, leading it to ameliorate oxidative events [38]. However, further experimental work needs to be conducted to examine this suggestion in the future.

Moreover, another metabolite that was upregulated in MSUD-affected newborns was L-arginine. Biologically, arginine has various body functions, including a protective role in disposing of toxic components such as ammonia and its related derivatives, as arginine can bind to ammonia to convert it into non-toxic compounds that are eventually excreted [39]. Potentially, the elevation of arginine in MSUD newborns may play a protective role, minimizing the toxic elevations of ammonia that have been previously observed in MSUD patients and have been associated with metabolic encephalopathy [40]. Thus, we anticipated that MSUD newborns might develop defensive mechanisms during the early neonatal stage of the disease, one of which is an increased level of arginine production. It is suggested that correlation studies be performed between the levels of arginine and ammonia in MSUD newborns’ DBS samples to examine the potential link between these two metabolites and help us understand the mechanism developed against the pathogenies of MSUD. Based on these amino acid-related findings, it seems that MSUD in the neonatal period leads to alterations in the structure and level of amino acids in patients. There is a need to study these amino acid alterations in patients of different ages who have or have not undergone treatment to understand these amino acid changes better.

### 3.4. Alterations in the Lipid Species in MSUD Newborns

Another observed metabolic alteration in our data is that various lipid species were disrupted, including the fatty acids, phospholipids, glycosphingolipids, ceramides, acylcarnitines, and glycerol derivatives. In our metabolomics data, LysoPI (16:0/0:0), Cer (d16:1(TXB2)), PE (15:0/18:3), PE-NMe (18:1/18:4), PI (18:1/PGJ2) and PA (2:0/18:2) were downregulated in MSUD newborns compared to controls. LysoPI is an endogenous ligand for G protein-coupled receptor 55(GPR55) and has various biological functions; however, its role in the central nervous system (CNS) is one that particularly affects microglial inflammatory responses. Microglia are immune cells with important functions in balancing the immune homeostasis of the CNS [41]. Additionally, Cer (d16:1/TXB2) is an oxidized ceramide, a member of the sphingolipids (SLs) or glycosylceramides. SLs are found in cell membranes, particularly in peripheral nerve cells and the cells found in the central nervous system. Impairments associated with sphingolipid metabolism are related to neurological syndromes [42]. The biosynthesis and catabolism of sphingolipids involve many intermediate metabolites and different enzymes. PE-NMe (15:0/18:3) is a monomethyl phosphatidylethanolamine, a glycerophospholipid part of phosphatidylcholine biosynthesis [43]. Phospholipids are key components of the cell lipid bilayer and are involved in metabolism and signaling. Oxidized phosphatidic acids, PA (2:0/18:2), belong to glycerolipids and can work as signaling molecules either by themselves or by indirectly interacting with other molecules. Those oxidized lipids are produced non-enzymatically through uncontrolled oxidation of free radicals, which are considered harmful to human health [44].

Furthermore, ganglioside GM1 (D18:0, 18:1) was downregulated in MSUD newborns compared to controls. Gangliosides are primarily considered the compositional components of the CNS glycome [45]. Gangliosides are glycosphingolipids made of glycan headgroups, which can engage with proteins or other glycans present on the same membrane, as well as with molecules on other cells and in the extracellular space, which results in modulation of cell signaling and communication [46,47]. GM1 exerts neuroprotective functions, specifically repairing the neuronal tissue after mechanical, biochemical, or toxic injuries [48]. Furthermore, our study showed various acylcarnitines (AC) alterations in MSUD newborns. Therefore, disrupted circulating AC may highlight the dysregulation of mitochondrial oxidation of lipids and upregulation of proinflammatory signals [49]. It could be that these alterations in lipid species worsen the phenotypes of MSUD in early life, including brain and nervous system damage.

### 3.5. Metabolites Involved in Oxidative Events in MSUD Newborns

Previously, it has been reported that MSUD patients have shown oxidative stress, which probably contributes to their neurological problems [50]. Regarding this state of oxidative stress state, our data showed that certain significant metabolites related to oxidative events are altered in MSUD. For example, in this study, glutathioselenol was upregulated in MSUD newborns. In the human liver, glutathioselenol reacts with glutathione to form hydrogen selenide ions, which are necessary to produce the seleno-proteins needed for biological systems, mostly as antioxidants [51,52]. Moreover, our findings showed that α-lipoic acid (ALA) was upregulated in MSUD newborns. ALA is a coenzyme of many multienzyme complexes located in the mitochondria. Mechanistically, ALA acts as an antioxidant by neutralizing free radicals and preventing oxidative damage to cells and tissues. It can work with other antioxidants such as vitamin C, vitamin E, and glutathione, enhancing the antioxidant defense system [53]. In addition, our data showed that N-acetylserotonin glucuronide (NASG) was downregulated in MSUD newborns. N-acetylserotonin glucuronide is derived from N-acetylserotonin (NAS), which controls intracellular redox states through the upregulation of enzymes involved in glutathione biosynthesis, enhancing the abundance of proteins involved in anti-oxidative defense [54]. Our metabolic finding suggested that the MSUD newborns potentially tried to develop defensive tools against the oxidative events using various mechanisms, such as glutathione- or ALA-mediated mechanisms, to reduce the health complications associated with MSUD. MSUD is a complicated case as it has many metabolic alterations, various phenotypes, and presentations, which enforce the identification of further diagnosis/screening biomarkers in addition to the standard markers used in NBS and genetic testing.

### 3.6. New Potential Metabolic Biomarkers/Pathways of MSUD

One of the well-known and definitive MSUD biomarkers is called “alloisoleucine”, which is used globally for MSUD diagnosis/screening during NBS [10]. It was detected in our untargeted metabolomics analysis of DBSs from MSUD newborns. Based on the biomarker analyses and our metabolic data, alloisoleucine was significantly elevated in MSUD newborns compared to healthy newborns, which makes untargeted metabolomics a trustworthy technique that could be used to validate the current standard method (NBS) and its MSUD markers. Our untargeted metabolomics analysis uncovered other new biomarkers, including methionine sulfoxide, LysoPI, and altered metabolic pathways such as ascorbate and aldarate metabolism, and pentose and glucuronate interconversions in MSUD newborns, potentially used as new additional biomarkers/pathways for MSUD. Particularly, Methionine sulfoxide was upregulated in MSUD newborns compared to healthy controls, and this could be attributed to the oxidative stress events in MSUD, making methionine sulfoxide a reliable biomarker for MSUD, needed for further validation studies. Also, LysoPI was selected as a decreased biomarker in MSUD, which merits further exploration.

As mentioned above, one of the most affected metabolic pathways in MSUD newborns is ascorbate and aldarate metabolism, compounds that are involved in the oxidative damage defense mechanism mediated by glutathione and other antioxidant molecules [55]. In addition, pentose and glucuronate interconversions were found in our data to be a highly impacted pathway in MSUD newborns. Glucuronate interconversions induce the glucuronidation process for the elimination of toxic molecules [56]. Predictably, in our study, MSUD newborns had impacted pentose and glucuronate interconversions, which may help remove the toxic substances that are a result of oxidative damage. However, further studies are required to examine the role of the glucuronidation process in MSUD.

These new metabolic biomarkers/pathways are very promising; they provide new insights into the field and should be considered additions to the diagnosis panel of MSUD in the future. However, a follow-up study is required to evaluate the discovered biomarkers’ reproducibility, stability, and performance and to validate the disrupted metabolic pathways in a bigger and independent cohort, considering an appropriate study design and FDR. Moreover, a prototype targeted analytical approach will be established for the newly discovered metabolic biomarkers using reference standard materials. Additionally, for accurate diagnosis, various biological samples, not only DBSs, must be used to examine sample-type specificity. Furthermore, a cohort of MSUD patients of different ages could be studied to determine whether these new metabolic biomarkers can only be used in the neonatal period or at other ages as well. All these suggested studies would generate more information about MSUD and develop more accurate approaches for its diagnosis/screening.

## 4. Materials and Methods

### 4.1. Ethical Approval

The Institutional Review Boards at King Faisal Specialist Hospital and Research Center (KFSHRC) in Riyadh, Saudi Arabia, reviewed and approved this study procedure (RAC No. 2160 027). The leftover samples submitted for routine clinical testing were waivered from any consents.

### 4.2. Participants’ Selection Criteria and Sample Collection

Forty-four DBS cards were collected from the Metabolomics Lab in the Center for Genomic Medicine at KFSHRC. The newborns positively screened with MSUD (*n* = 22) were confirmed biochemically and genetically and included in this study with healthy controls (*n* = 22). All healthy controls were age- and gender-matched with patients. MSUD newborns were not diagnosed with any other disease, such as IEM disorders, and less than 14 days old were included in this study. Originally, these samples were collected from newborns’ heel pricks dripped from 903 Protein Saver cards (Whatman, Piscataway, NJ, USA). Then, after being dried, the DBS cards were stored at 4 °C for later biochemical and metabolomics analyses. The initial newborn screening was performed with tandem mass spectrophotometry using MassChrom kits (cat# 55000, ChromSystems, Munich, Germany), a CE-Marked diagnostic Kit available commercially. This screening test was performed routinely in our lab for clinical purposes, where the kit contains mobile phase, quality control samples, internal standard solutions labeled for the key amino acids, and acylcarnitine. In this study, this kit measured the leucine, isoleucine, valine, and MSUD markers as part of our routine laboratory practice.

### 4.3. Chemicals

The LC-MS graded chemicals methanol (MeOH), acetonitrile (ACN), deionized water (dH_2_O), and formic acid were purchased from Fisher Scientific Company (Ottawa, ON, Canada). The reference materials used as internal standards were purchased from Sigma (Ottawa, ON, Canada).

### 4.4. Sample Preparation

Metabolites were extracted from the DBS of MSUD and newborn healthy controls. One punch of DBS with a size of 3.2 mm was distributed in 96 V-shaped plate wells. Then, the punch was immersed in 250 μL of (dH_2_O: MeOH: ACN) (20:40:40%) as an extraction solvent. The samples were vortexed in a ThermoMixer (Eppendrof, Hamburg, Germany) at 600 rpm, 25 °C, for 2 h. Subsequently, the samples were spun down at 16,000 rpm, 4 °C, for 10 min. The supernatants were transferred into new 96-well V-shaped plates, the punches were discarded, and the samples were evaporated in a Speed-Vac (Thermo Fischer, Christ, Germany) [57].

### 4.5. LC-HRMS Metabolomics Analysis

All dried extracted samples were reconstituted in 50% mobile phase A (0.1% formic acid in deionized water) and mobile phase B (0.1% formic acid in (1:1) (*v*/*v*) MeOH and ACN) for an LC-MS metabolomics analysis. Initially, 5 μL of the reconstituted sample was introduced to the inlet technique, where the metabolites were separated in a reversed-phase liquid chromatography with Waters ACQUITY UPLC XSelect C18 (100 × 2.1 mm × 2.5 μm) column (Waters Ltd., Elstree, UK). The mobile phase flow rate was set to 300 μL/min, and the column was maintained at 55 °C while the sample was stored at 4 °C in the autosampler. Mobile phases A and B were pumped in a gradient mode as follows: 95–5% A (0–16 min), 5% A (16–19 min), 5–95% A (19–20 min), and 5–95% A (20–22 min). The eluted molecules from the column were ionized in the electrospray ionization source (ESI) at positive and negative modes. The gas phase ions were subjected to Xevo G2-S QTOF mass spectrometer (Waters Ltd., Elstree, UK) separation based on their *m*/*z*. The MS source temperature was fixed at 150 °C, the desolvation temperature was set at 500 °C, and the capillary voltages were kept at 3.20 kV or 3 kV for ESI+ and ESI− modes, respectively. The cone gas flow was 50 L/h, the desolvation gas flow was 800 L/h, and the cone voltage was 40 V. The collision energies for the low and high functions were set to off and 10–50 V, respectively, in the MSE data-independent acquisition (DIA) mode. As recommended by the vendor, the mass spectrometer was calibrated with sodium formate (100–1200 Da) in both ionization modes. The lock spray mass compound, MS leucine-enkephaline (an external reference to the ion *m*/*z* 556.2771 in positive mode and 554.2615 in negative mode), was constantly injected, which is responsible for switching between the sample and the reference for every 45 and 60 s in both modes, scan time was 0.5 s, the flow rate was 10 μL/min, and collision energy was 4 V and 30 V for the cone, respectively. The DIA data were gathered in continuum mode with Masslynx™ V4.1 Software (Waters Inc., Milford, MA, USA). Quality control samples (QCs) were performed gently by collecting a single 3.2 mm punch from each study sample and pooling them for extraction. After that, they were introduced to the instrument randomly to validate the system’s stability [58]. After that, they were analyzed following the routine protocol. The acceptance criteria were to have all the QC samples separated from the other study groups, clustered together, and use their Relative standard deviations (RSD%) < 40%.

### 4.6. Metabolomics Data Processing and Statistical Analyses

**Scheme 1:**
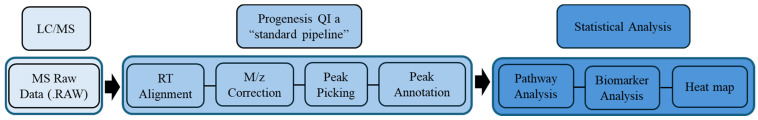
The workflow of data analyses performed in this study.

The raw MS data were processed using a standard pipeline, retention time alignment, mass-to-charge ratio (*m*/*z*) correction followed by the compound peak picking. Compound signals were selected based on the quality of the peaks that were selected, where the noise peaks were excluded outside the RT range of 0.5–20 min, and fragmentation ions with intensity below 0.2% were included using Progenesis QI v.3.0 software (Waters Technologies, Milford, MA, USA; Scheme 1). Multivariate statistics was applied using MetaboAnalyst (v.5.0; McGill University, Montreal, QC, Canada; http://www.metaboanalyst.ca, accessed on 20 June 2023) [59]. All the imported data groups (compounds’ names and their raw abundance information) were normalized by the median and were Pareto scaled and log-transformed, and these were used to create PLS-DA and OPLS-DA models. The generated OPLS-DA model was measured through (R^2^Y) and (Q^2^) values, representing the model’s fitness and predictive ability, respectively [60]. A univariate analysis was applied with Mass Profiler Professional (MPP) v.15.0 software (Agilent, Santa Clara, CA, USA). A volcano plot was used to uncover significantly changed mass features based on a moderated *t*-test, cut-off: *p* < 0.05, fold change 1.5. Compared to the controls, heatmap analysis for altered features in MSUD newborns was performed using the Pearson distance measure according to the Pearson similarity test [61]. Pathway and biomarker analyses, linked with MSUD-linked biomarkers, and receiver operating characteristic (ROC) curves were created using the PLS-DA approach in the MetaboAnalyst v 5.0 for globe analysis to specify potential biomarkers.

### 4.7. Metabolite Identification (Peak Annotation)

All the statistically significant features between the study groups were selected using Progenesis QI v.3.0 software (Waters Technologies, Milford, MA, USA) for peak annotation. The precursor and product ions were annotated based on accurate mass, fragmentation pattern, and isotopic distributions in the Human Metabolome Database (HMDB) with a 5 ppm mass error [62]. Exogenous metabolites, such as food additives, pharmaceuticals, and exposomes, were removed from the finalized list.

## 5. Conclusions

This study has presented valuable information about the metabolic alterations in amino acids, lipids, and other molecules and the potentially induced oxidative events in the neonatal period of MSUD. Additionally, this study revealed its capability to measure the standard MSUD biomarker “alloisoleucine” and uncovered new metabolic biomarkers/pathways (methionine sulfoxide; LysoPI; ascorbate and aldarate metabolism, and pentose and glucuronate interconversions) that will be able to be used in its diagnosis/screening in the near further, after further validation studies. These future studies will strengthen our findings and help improve the accuracy of the current diagnostic tools, NBS and genetic tests, for better health outcomes and disease treatments.

## Figures and Tables

**Table 1:** Demographic and clinical features of MSUD newborns and healthy controls.

Demographic and Clinical Features	MSUD *(n* = 22)		CTRL *(n* = 22)		*p*-Value
	Mean	SEM	Mean	SEM	
Age (Day)	7.63	3.07	7.72	3.18	0.9421
Female (%)	41	NA	41	NA	NA
Male (%)	59	NA	59	NA	NA
Xleucine (Cutoff: <245 µM)	581	431.97	<245 µM	NA	0.005 **
Valine (Cutoff: <290 µM)	425.15	149.02	<290 µM	NA	0.0042 **

For statistical analyses, an independent student *t*-test was conducted. Mean ± SEM expresses data. Student’s *t*-test: Two-tailed was applied (** *p* < 0.01). MSUD Maple syrup urine disorder; CTRL Healthy control; SEM standard error of the mean; NA not applicable.

## Data Availability

The raw data of this study were deposited at Metabolomics Workbench on 18 July 2023 and can be accessed under accession number ST002750.

## Supplementary Materials

The supporting information can be downloaded at: https://www.mdpi.com/article/10.3390/ijms25115720/s1.
