# Supplementary material for: Exploratory Untargeted Metabolomics of Dried Blood Spot Samples from Newborns with Maple Syrup Urine Disease
Source: Int J Mol Sci. 2024 May 24;25(11):5720. doi: 10.3390/ijms25115720 (PMC11171634; doi:10.3390/ijms25115720)
Supplement: Supplementary file 1 [file ijms-25-05720-s001.zip › Figure S1;Hierarchical Entity Tree on groups (Non-averaged).pdf]

Hierarchical Entity Tree on groups (Non-averaged) Report

Experiment : newborn MSUD G210 HEATMAP
Active entity list : All Entities

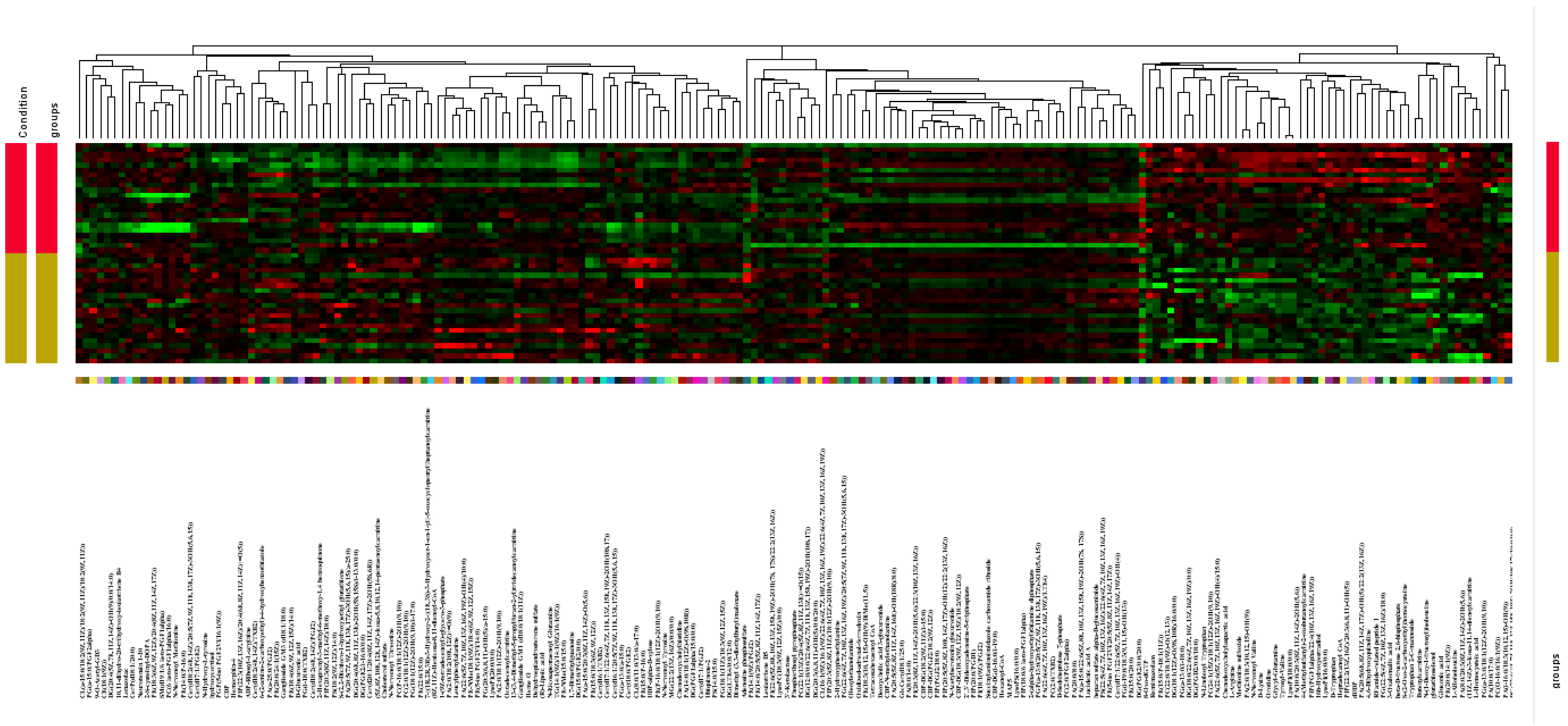

Color range

-3.9 -1.9 0 1.9 3.9

Condition dataset colors Condition

[N_Ctrl]	[N_MSUD]

Condition dataset colors groups

N_Ctrl	N_MSUD

Entity color by Alignment Value

(11Z,14Z)-3-Icosa-11,14-die	(5Z,8Z,10E,12E,14Z)-Icosa-	(8Z,11Z)-Henicosa-8,11-die	(R)-Lipoic acid	1,7-Dimethylguanosine
1-(9Z-tetradecenoyl)-glycero	10,11-dihydro-20-trihydroxy-	13-(3,4-Dimethyl-5-propylfur	16b-Hydroxyestradiol	2',3'-Dideoxyadenosine-5-tri
2-(alpha-hydroxyethyl)thiami	2-Hexaprenyl-3-methyl-6-me	2-Hydroxyphenethylamine	2-S-cysteinyl-DOPA	3'-Ketolactose
3-Oxalomalic acid	4,6-Dihydroxyquinoline	4-(Methylsulfanyl)-2-oxobuta	6-(2-amino-2-carboxyethyl)-	7-[(1R,2R,3R)-3-Hydroxy-2-[
8-Oxo-dGTP	9-O-Acetyl-GD3	ADP-Ribosyl-L-arginine	Adenosine phosphosulfate	Butenylcarnitine
CDP	CDP-DG(18:2(9Z,11Z)/i-15:	CDP-DG(18:3(9Z,12Z,15Z)/	CDP-DG(PGJ2/18:2(9Z,12Z	CDP-DG(i-16:0/i-19:0)
CDP-N-methylethanolamine	CE(18:1(9Z))	CL(16:1(9Z)/16:1(9Z)/22:6(4	CL(8:0/11:0/13:0/a-17:0)	CL(a-15:0/18:2(9Z,11Z)/18:2
Cer(d16:1/20:5(7Z,9Z,11E,1	Cer(d16:1/TXB2)	Cer(d17:1/22:6(4Z,7Z,11E,1	Cer(d17:1/22:6(5Z,7Z,10Z,1	Cer(d17:1/PGE2)
Cer(d17:1/PGJ2)	Cer(d18:2(4E,14Z)/20:5(7Z,	Cer(d18:2(4E,14Z)/PGJ2)	Cer(d18:2(4E,14Z)/TXB2)	Cer(d20:1/20:4(8Z,11Z,14Z,
Cer(t18:0/PGE2)	CerP(d18:1/20:0)	Chenodeoxycholylaspartic a	Chenodeoxycholylhistidine	Cholesterol sulfate

Description

Created from Advanced Analysis operation: Clustering:
Entity List: All Entities
Interpretation: groups (Non-averaged)
Experiment: newborn MSUD G210 HEATMAP
Clustering Algorithm: Hierarchical
Clustered By: Normalized intensity values
Clustered On: Entities
Similarity Measure: PearsonCentered
Linkage Rule: Average

Sample Parameter Plots

Heat Map

groups

N_Ctrl

N_MSUD
